# Impact of Translocator Protein 18 kDa (TSPO) Deficiency on Mitochondrial Function and the Inflammatory State of Human C20 Microglia Cells

**DOI:** 10.3390/cells12060954

**Published:** 2023-03-21

**Authors:** Stefanie Bader, Thea Würfel, Tatjana Jahner, Caroline Nothdurfter, Rainer Rupprecht, Vladimir M. Milenkovic, Christian H. Wetzel

**Affiliations:** Department of Psychiatry and Psychotherapy, University of Regensburg, 93053 Regensburg, Germany

**Keywords:** translocator protein 18 kDa, mitochondria, inflammation, CRISPR/Cas9, respirometry, metabolic profile, glycolysis, OxPhos, calcium imaging

## Abstract

Microglia are the resident immune cells of the central nervous system. Upon stimulus presentation, microglia polarize from a resting to an activated state. Microglial translocator protein 18 kDa (TSPO) is considered a marker of inflammation. Here, we characterized the role of TSPO by investigating the impact of TSPO deficiency on human microglia. We used TSPO knockout (TSPO−/−) variants of the human C20 microglia cell line. We found a significant reduction in the TSPO-associated protein VDAC1 in TSPO−/− cells compared to control cells. Moreover, we assessed the impact of TSPO deficiency on calcium levels and the mitochondrial membrane potential. Cytosolic and mitochondrial calcium concentrations were increased in TSPO−/− cell lines, whereas the mitochondrial membrane potential tended to be lower. Assessment of the mitochondrial DNA copy number via RT-PCR revealed a decreased amount of mtDNA in the TSPO−/− cells when compared to controls. Moreover, the metabolic profiles of C20 cells were strongly dependent on the glycolytic pathway. However, TSPO depletion did not affect the cellular metabolic profile. Measurement of the mRNA expression levels of the pro-inflammatory mediators revealed an attenuated response to pro-inflammatory stimuli in TSPO-depleted cells, implying a role for the TSPO protein in the process of microglial polarization.

## 1. Introduction

Microglia are tissue-resident macrophages of the central nervous system (CNS). As resident immune cells within the brain, microglia fulfill a wide range of tasks, ranging from the detection of pathogens, mediation of pro-inflammatory reactions, antigen presentation, and conveying neuroprotective properties to the promotion of neuronal regeneration [1]. Upon detection of potentially dangerous stimuli or inflammatory signals, microglia undergo functional and morphological changes into an activated state, being either pro- or anti-inflammatory, which is dependent on the received stimuli [2]. The pro-inflammatory or M1 microglial state is also referred to as the classically activated phenotype and is mainly triggered by lipopolysaccharides (LPS), reactive oxygen species (ROS), or inflammatory factors such as interferon-γ (INFγ) or tumor necrosis factor alpha (TNFα) [3]. However, it can also be induced by hypoxia or the detection of cellular debris due to injury to the surrounding tissue [4]. M1 microglia can initiate adaptive immune responses by presenting antigens using MHCII receptors, the release of pro-inflammatory cytokines such as TNFα, interleukins 1β (IL1β), -12A (IL12A), -6 (IL6), and -8 (IL8), as well as the upregulation of proteins related to the production of nitric oxide (NO), such as the inducible nitric oxide synthase (iNOS, NOS2). It also regulates the expression of mitochondrial proteins, such as the translocator protein 18 kDa (TSPO), as well as mitochondrial (and therefore energetic) function [5,6]. M1 microglia undergo a so-called “metabolic switch” when activated, thereby changing the main cellular energy production from oxidative phosphorylation to glycolysis by upregulating transporters that facilitate glucose uptake, like GLUT1. This is paralleled by a change in mitochondrial parameters like oxygen consumption rate or morphological constitution [7].

The translocator protein 18 kDa (TSPO) is a highly conserved multifunctional protein residing in the outer mitochondrial membrane (OMM) [8]. It is expressed to varying degrees in multiple tissues, with the highest levels of expression found in steroidogenic tissues and relatively low expression in the central nervous system. In the brain, TSPO can be detected predominantly in microglia and reactive astrocytes [9], where its expression is upregulated in the context of inflammation, neurodegeneration, and malignant neoplasia [10,11]. Although the role of TSPO in de novo steroidogenesis was questioned in view of various genetic TSPO deletion models [12,13,14,15], our recent studies support a role of TSPO expression as well as of TSPO ligands in the regulation of pregnenolone synthesis in mouse BV-2 and human C20 microglia cells [16,17,18]. In addition its suggested role in cholesterol transport and steroid synthesis [15,19,20], TSPO has been shown to be involved in the regulation of various cellular and mitochondrial functions. The multifunctional properties of TSPO also affect mitochondrial bioenergetics (oxidative phosphorylation, OxPhos) and metabolism [17,21], beta-oxidation of fatty acids [22], the production of ROS [23], and Ca^2+^ homeostasis [17,24]. Moreover, TSPO is also involved in regulating cellular downstream processes such as proliferation, survival, and apoptosis [10,25].

In the present study, using CRISPR/Cas9, we newly generated TSPO−/− and control lines of established human microglia C20 cells. We investigated the effect of TSPO deficiency on mtDNA copy number, cytosolic and mitochondrial Ca^2+^ levels, and the mitochondrial membrane potential. Since current hypotheses argue for a structural and functional interaction of TSPO with the voltage-dependent anion channel (VDAC1) [26], we also assessed VDAC1 expression in TSPO−/− and control cells. Moreover, we investigated the metabolic profile of TSPO-deficient and control C20 cells by evaluating their dependence on oxidative phosphorylation or glycolysis under basal or an activated condition achieved by treating the cells with a cytokine cocktail of TNFα, IL1β, and INFγ [27]. The activated state of the treated microglia cells was verified by analyzing the mRNA expression profile of pro-inflammatory cytokines.

## 2. Materials and Methods

### 2.1. Cell Lines and Culture Conditions

Human microglia C20 cells [28] were grown in Dulbecco’s Modified Eagle’s Medium/Nutrient Mixture F-12 Ham (Sigma Aldrich, Taufkirchen, Munich, Germany), supplemented with 10% fetal calf serum (FCS), 2 mM L-glutamine, and 10,000 U/mL penicillin-streptomycin at 37 °C in humidified air with 5% CO_2_, and the medium was changed three times a week.

### 2.2. CRISPR/Cas9-Mediated TSPO Knockout

To inactivate the TSPO gene in C20 cells, the CRISPR-Cas9 system was adapted from [29]. Targeting sequences were designed using the Web-based tool CRISPOR (http://crispor.tefor.net/, accessed on 27 November 2020). Two independent target sequences for the human TSPO gene were used: #116: 5′-TCCCGCTTTGTCCACGGCGAGGG-3′, and #126: 5′-TCCACGGCGAGGGTCTCCGCTGG-3′. The DNA sequences were synthesized (Metabion, Planegg-Martinsried, Munich, Germany) and separately cloned into the vector pSpCas9(BB)-2A-GFP. PX458 (a gift from Feng Zhang, Addgene plasmid # 48138, Addgene, Cambridge, MA, USA) drives the expression of Streptococcus pyogenes Cas9, GFP, and the chimeric guide RNA in mammalian cells. C20 cells were transfected with the recombinant plasmids using jetOptimus (Polyplus, New York, NY, USA). Twenty-four hours after transfection, single GFP-positive cells were sorted by flow cytometry to allow single-colony formation. After 7 days, single clones were collected, and the successful knockout was confirmed by Western blotting using anti-rabbit TSPO antibodies and sequencing of genomic DNA. All experiments were performed on two independent isogenic TSPO-deficient clones and two isogenic control cell lines.

### 2.3. Western Blotting

Whole cell protein samples were sonicated and boiled in RIPA buffer, and total protein was quantified using a micro-BCA colorimetric assay (Pierce, Thermo Fischer Scientific, Darmstadt, Germany). Protein samples were separated by SDS-polyacrylamide gel electrophoresis on 15% gels and subsequently transferred onto a nitrocellulose membrane (Amersham™ Protran 0.45 NC, GE Healthcare Life Sciences, Chicago, IL, USA). TSPO and VDAC1 proteins were detected using rabbit anti-TSPO (ab109497) and mouse anti-VDAC1 (ab186321) antibodies, both from Abcam, Cambridge, UK, respectively. Beta-1 tubulin antibody (clone E7, Developmental Studies Hybridoma Bank, Iowa, IA, USA) was used as a loading control. VDAC1 band densities were quantified using ImageJ2 Version 2.9.2. [30] and normalized to beta-1 tubulin.

### 2.4. RNA Isolation, Reverse Transcription, and Quantitative Real-Time RT-PCR of Inflammatory Markers

Total RNA was extracted using the RNA Plus Kit (Macherey-Nagel, Düren, Germany) according to the manufacturer’s instructions. First-strand cDNA synthesis from 1 μg of total RNA was performed with the QuantiTect Reverse Transcription Kit (Qiagen, Hilden, Germany). Quantitative RT-PCR experiments were performed with the Rotor-Gene-Q machine (Qiagen, Hilden, Germany) using the 1× Takyon SYBR Master Mix (Eurogentec, Köln, Germany) and specific intron-spanning primers, listed in Table 1. Measurements were performed in triplicate, and results were analyzed with Rotor-Gene-Q software version 2.3 (Qiagen, Hilden, Germany), applying the ΔΔCt method for relative quantification.

### 2.5. mtDNA Copy Number

For the quantification of the mtDNA copy number, primers for the mitochondrial gene mt-TL1 and the nuclear gene B2M are used. Primer sequences are listed in Table 1. The mtDNA copy number was assessed using three biological replicates for each cell line. Each biological replicate was measured in triplicate in two separate runs. Analysis was performed using Rotor-Gene-Q software version 2.3 (Qiagen, Hilden, Germany), by relating the ΔΔCt of genomic and mitochondrial genes of each sample to the overall efficiency of the primers E multiplied by two to account for the diploid nDNA copies per cell.

2 × E^−ΔΔCt^

### 2.6. ATP Content

ATP concentration was measured using CellTiter-Glo Reagent (Promega, Walldorf, Germany) according to the manufacturer’s instructions. Briefly, 1 × 10^5^ cells were collected and stored at −20 °C if necessary. The cell pellets were resuspended in 500 µL of PBS and heated at 100 °C for 2 min in order to remove ATPases. Subsequently, absorption was measured using a VarioScan (Thermo Fischer Scientific, Darmstadt, Germany) with an integration time of 1 s. The relative light unit (RLU) generated by the SkanIT Software (Version 2.4.5.) (Thermo Fischer Scientific, Darmstadt, Germany) can be calculated to the actual ATP concentrations with the help of the ATP standard curve. ATP values were normalized to the total protein content using a micro-BCA assay.

### 2.7. Mitochondrial Membrane Potential

A quantity of 2.5 × 10^4^ C20 wild-type or TSPO knockout cells were seeded on sterile glass coverslips (diameter 25 mm), placed in 6-well plates, and grown overnight in Dulbecco’s Modified Eagle’s Medium/Nutrient Mixture F-12 Ham at 37 °C, humidified air, and 5% CO_2_. Cells were loaded with 200 nM JC-1/Pluronic (Life Technologies, Darmstadt, Germany) in Opti-MEM (Life Technologies, Darmstadt, Germany) for 30 min at 37 °C in humidified air with 5% CO_2_. For imaging, coverslips were washed with assay buffer (140 mM NaCl, 5 mM KCl, 1.8 mM CaCl_2_, 1 mM MgSO_4_, 10 mM glucose and 10 mM HEPES) and mounted in a chamber on the inverted microscope (ZEISS Observer Z.1, Jena, Germany). A Lambda DG4 high-speed wavelength switcher (Sutter Instruments, Novato, CA, USA) allowed the excitation of JC-1 at 480/36 nm. The emitted light was filtered at 537/42 nm and 620/60 nm for green or red fluorescence, respectively, and finally detected by a CCD camera (AxioCam MRm, ZEISS, Jena, Germany). Mitochondrial membrane potential was analyzed as a ratio of red versus green fluorescence intensity in regions of interest, drawn over selected cells in the visual field using ImageJ [30]. The macro used for background subtraction and analysis will be provided on request.

### 2.8. Cytosolic Ca^2+^ and Mitochondrial Ca^2+^ Levels

A quantity of 2.5 × 10^4^ C20 wild-type or TSPO knockout cells were grown on sterile glass coverslips (diameter 25 mm) overnight and loaded with 2 µM Fura-2/AM and 2 µM Rhod2/AM (Life Technologies, Darmstadt, Germany) in opti-MEM for 30 min at 37 °C and 5% CO_2_. The experiments were performed using a ZEISS live cell imaging setup (ZEISS, Jena, Germany). Fura-2/AM-loaded cells (2 µM, 30 min at 37 °C) were illuminated with light of 340 or 380 nm (BP 340/30 HE, BP 387/15 HE) using a fast wavelength switching and excitation device (Lambda DG-4, Sutter Instrument, Novato, CA, USA). Fluorescence was detected at 510 nm (BP 510/90 HE and FT 409) using an AxioCam MRm LCD camera (ZEISS, Jena, Germany). Fluorescence in Rhod2/AM-loaded cells was excited at 545/25 nm, and images of the emitted fluorescence were collected using a 605/70 nm emission filter using ZEN 2012 software (ZEISS, Jena, Germany), which was used to control the hardware and acquire data. Cells were selected as ROIs in the visual field using FIJI/ImageJ [30] as described for the procedure used to assess the MMP. The macro used for background subtraction and analysis will be provided on request.

### 2.9. Pharmacology/Pro-Inflammatory Stimulation

To induce microglial activation, TSPO−/− and control C20 cell lines were treated with a mixture of human tumor necrosis factor α (TNFα), Interleukin 1β (IL1β), and Interferon-γ (INFγ). All cytokines were diluted in PBS + 1% bovine serum albumin (BSA) at a concentration of 10 μg/mL (TNFα and IL1β) or 20 μg/mL (INFγ) and stored in aliquots at −80 °C to avoid multiple thaw/freeze cycles. For treatment, the cytokines were thawed and diluted in normal media to an end concentration of 1 ng/mL each. The cells were then treated with the cytokine mixture for 16 h. After treatment, the cells were harvested, pelleted, and frozen at −20 °C until further use.

### 2.10. Metabolic Profile

To study the cells’ metabolism in basal state and after pro-inflammatory stimulation, oxidative phosphorylation or glycolysis were blocked with either Antimycin A or 2-Deoxy-D-glucose, respectively, and the cell survival was measured via a resazurin assay. Antimycin A was used at concentrations of 10 μM, 7.5 μM, 5 μM, 2.5 μM, and 1 μM, while 2-Deoxy-D-glucose was used at concentrations of 300 mM, 100 mM, 50 mM, 25 mM, and 5 mM in triplicates each. For each cell line, cells were seeded onto a 96-well plate, with roughly 5000 cells per well. Cells were grouped into three subcategories: basal (1), stimulated for 16 h (2), and stimulated for 40 h (3). Stimulation was performed with 1 ng/mL of the cytokine cocktail containing TNFα, IL1-β, and INFγ. After 16 h of incubation with the cocktail (2) or basal media (1), the media was removed and replaced by media containing the inhibitor at the concentrations described above. For subgroup (3), the cytokine cocktail was added additionally at 1 ng/mL. After 20 h of incubation with inhibitors +/- cytokines, 10% resazurin was added to each well and left to incubate for four hours at 37 °C. The fluorescent signal was then measured at 590 nM using the VarioScan (Thermo Fischer Scientific, Darmstadt, Germany).

### 2.11. Statistical Analysis

Data were expressed as the mean ± standard error of the mean. Statistical analysis was performed with GraphPad Prism 9 (GraphPad Software, Boston, MA, USA). Statistical significance was assessed using the independent samples *t*-test, Mann–Whitney U test, or ANOVA, combined with post hoc tests for multiple comparisons. Results were regarded as statistically significant for *p* < 0.05.

## 3. Results

In order to deeply characterize the impact of TSPO expression on mitochondrial function and cellular physiology in human microglia cells, we used the established human C20 microglia cell line [28]. We generated several isogenic TSPO-deficient clones from one parental cell line by means of CRISPR/Cas9-mediated gene knockout using two distinct guide sequences. A different batch of C20-derived cell lines was already used in earlier studies to investigate the impact of TSPO-expression on various cellular functions [17].

### 3.1. Mitochondrial DNA Copy Number

**Figure 1:**
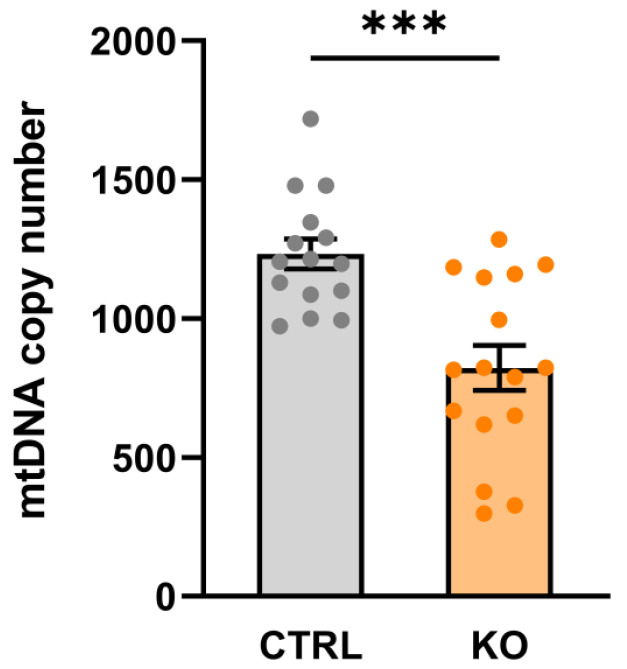
mtDNA copy number in TSPO−/− and control C20 cell lines. Data are presented as single values of six RT-PCR ± SEM (n = 3 biological replicates). The mtDNA copy number is significantly decreased in the TSPO-KO compared to the control cells (stars indicate significance *p* = 0.0003; Welch’s corrected *t*-test).

Mitochondria possess their own DNA (mtDNA, 16.5 kb), which codes for 13 mitochondrial proteins, 22 tRNA molecules, and 2 rRNA molecules. Cells contain up to thousands of circular mtDNA molecules, and the amount of mtDNA per cell, i.e., the mtDNA copy number, corresponds to the number of mitochondria (mitochondrial mass) per cell. We found that TSPO−/− cells contained significantly less mtDNA compared to their controls (mtDNA copy number in TSPO−/− 822.1 ± 80.37, control 1232 ± 53.78, *p* = 0.0003; Welch’s corrected *t*-test) (Figure 1).

### 3.2. VDAC1 Expression

**Figure 2:**
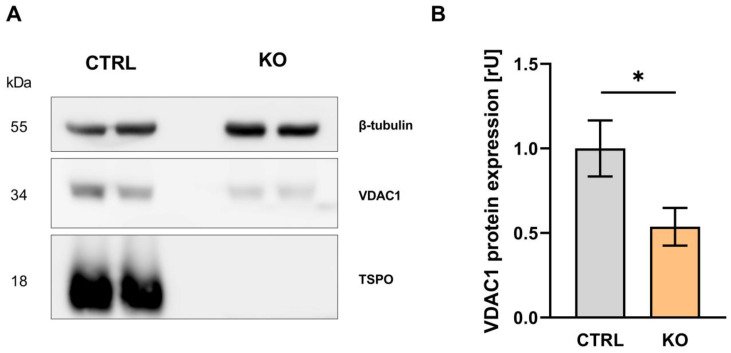
VDAC1 expression in C20 human microglia, TSPO−/−, and control cell lines. (**A**) Western blot depicting the housekeeper gene β-tubulin at 55 kDa, VDAC1 at 34 kDa, and TSPO at 18 kDa. The TSPO−/− cell lines show no visible band at 18 kDa. (**B**) Relative expression of VDAC1 normalized to β-tubulin expression in control and TSPO−/− cell lines. The relative expression of VDAC1 is significantly reduced in the KO cell lines (mean ± SEM, n = 3 biological replicates; star indicates significance *p* < 0.05).

According to current hypotheses, TSPO is a highly interacting protein that may function as a molecular hub to orchestrate and regulate the function and expression of proteins associated with a multimeric complex. VDAC1-expression in TSPO-deficient C20 cells is reduced to 54% of control (*p* = 0.0204; unpaired *t*-test) (Figure 2), supporting the idea of a functional and structural interaction between both proteins. In the context of altered VDAC1-expression, TSPO-deficiency directly or indirectly affects important physiological parameters of mitochondrial function.

### 3.3. Intracellular Ca^2+^ Levels

**Figure 3:**
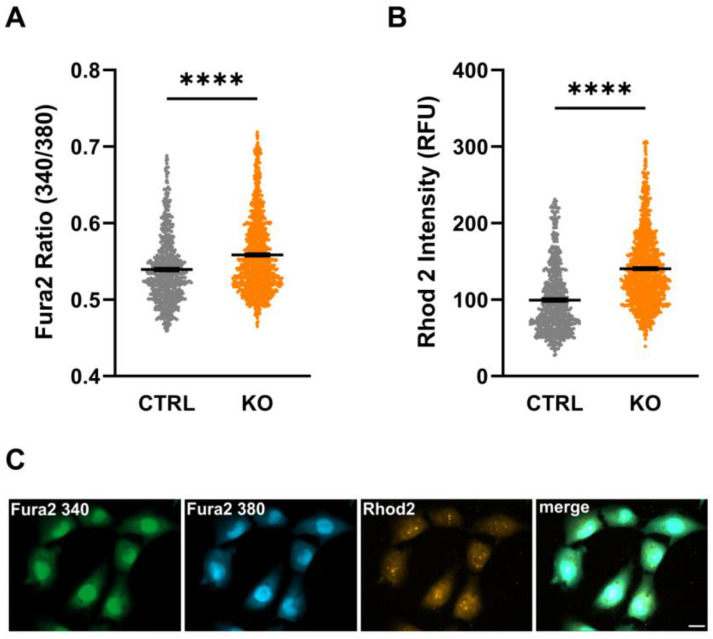
Cytosolic and mitochondrial Ca^2+^ levels in control and TSPO-deficient human C20 microglia cells. Impact of TSPO deficiency on cytosolic (**A**) and mitochondrial Ca^2+^ levels (**B**). The Fura-2 ratio (**A**) and Rhod-2 fluorescence intensity (**B**) were significantly increased in TSPO−/− cells compared to their controls. Shown are single values and the mean of n = 3 experiments. (**C**) Exemplary image indicating Fura-2/AM and Rhod-2/AM loaded cells. Scale bar: 20 µm. Stars indicate significance *p* < 0.0001.

Assessment of cytosolic as well as mitochondrial Ca^2+^ levels using the fluorescent Ca^2+^-sensitive dyes Fura-2/AM and Rhod-2/AM, respectively, revealed a significantly increased Fura-2 ratio (cytosolic Ca^2+^: 0.539 ± 0.002 vs. 0.558 ± 0.002; *p* < 0.0001, n = 3 experiments) and Rhod-2 intensities (mitochondrial Ca^2+^: 99.42 ± 1.75 vs. 140.4 ± 1.46; *p* < 0.0001, n = 3 biological replicates, Mann–Whitney U test) in TSPO-deficient C20 cells compared to their controls (Figure 3). These data indicate increased cytosolic and mitochondrial Ca^2+^ levels in TSPO−/− cells, possibly affecting numerous Ca^2+^-sensitive processes (also compare [17]).

### 3.4. Size of C20 Microglia Cells

**Figure 4:**
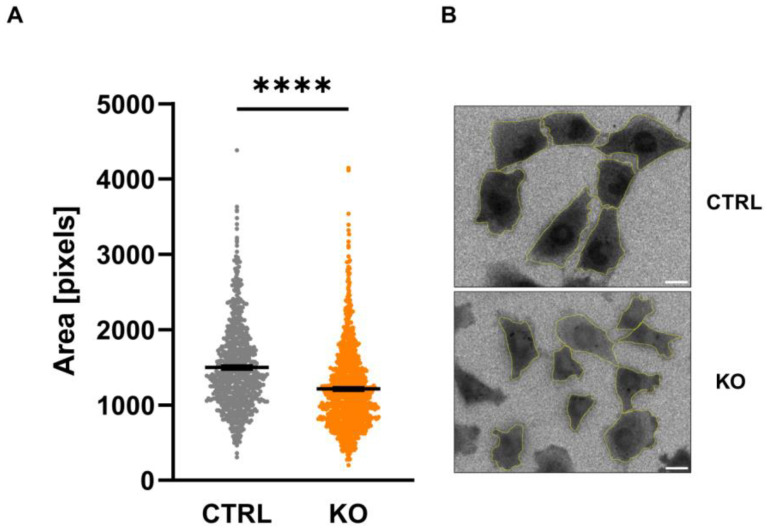
Size of C20 microglia cells. The area of C20 microglia cells was assessed by counting the number of pixels visualized after loading the cells with Fura-2 (**A**) (CTRL 1498 ± 21.86 vs. KO 1213 ± 15.97; *p* < 0.0001; n = 3; Mann–Whitney U test). Exemplary image indicating Fura-2-loaded cells (ratio image), and the analyzed region of interest is shown in (**B**). Scale bar: 20 µm. Stars indicate significance.

Analysis of the size of the C20 microglia cells by assessing the fluorescent area after loading the cells with Fura-2 revealed a significantly smaller cell size in TSPO−/− cells (Figure 4).

### 3.5. Mitochondrial Membrane Potential

**Figure 5:**
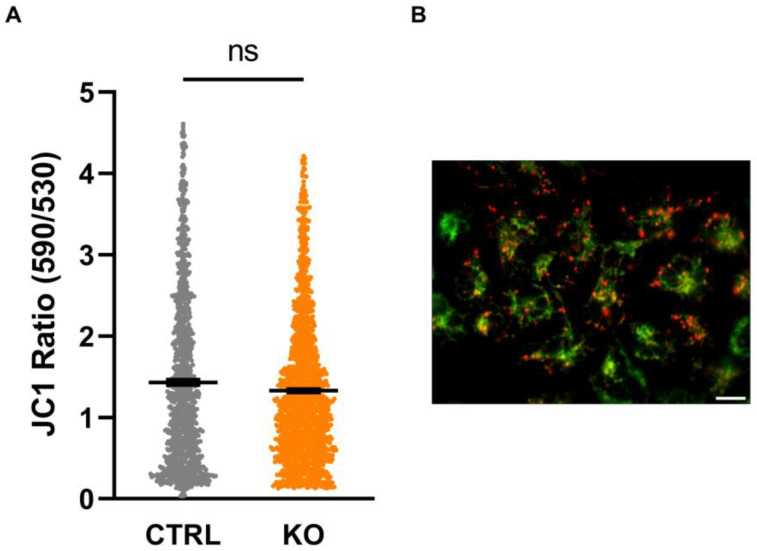
Mitochondrial membrane potential (MMP). The cationic dye JC-1 is attracted by the negative membrane potential of the mitochondria and forms red fluorescing aggregates in highly energized (strongly hyperpolarized MMP) mitochondria. JC-1 aggregates dissociate into green, fluorescent monomers upon reduction (depolarization) of the MMP. Thus, the ratio can be used to estimate the polarity of the MMP. (**A**) Shown are single values and the mean of n = 3 experiments (CTRL 1.43 ± 0.032 vs. KO 1.33 ± 0.022; n = 3 biological replicates; Mann–Whitney U test). (**B**) Exemplary image indicating JC-1-loaded mitochondria. Scale bar: 20 µm. ns indicates a non-significant difference.

The mitochondrial membrane potential (MMP) as an integrative measure of bioenergetic capacity is established by the translocation of protons across the inner mitochondrial membrane (IMM) associated with the transport of electrons along the respiratory chain complexes, which results in a significant proton motive force. On the other hand, this proton gradient is diminished by the backflow of protons into the matrix when driving the ATP synthase, which generates ATP from ADP and phosphate. We found no difference in the MMP, indicated by the ratio of the fluorescence of JC-1 aggregates (indicating a negative MMP resulting in red fluorescence) over JC-1 monomers (less negative MMP, green fluorescence) between TSPO-deficient cells and controls (Figure 5).

### 3.6. Metabolic Profile

**Figure 6:**
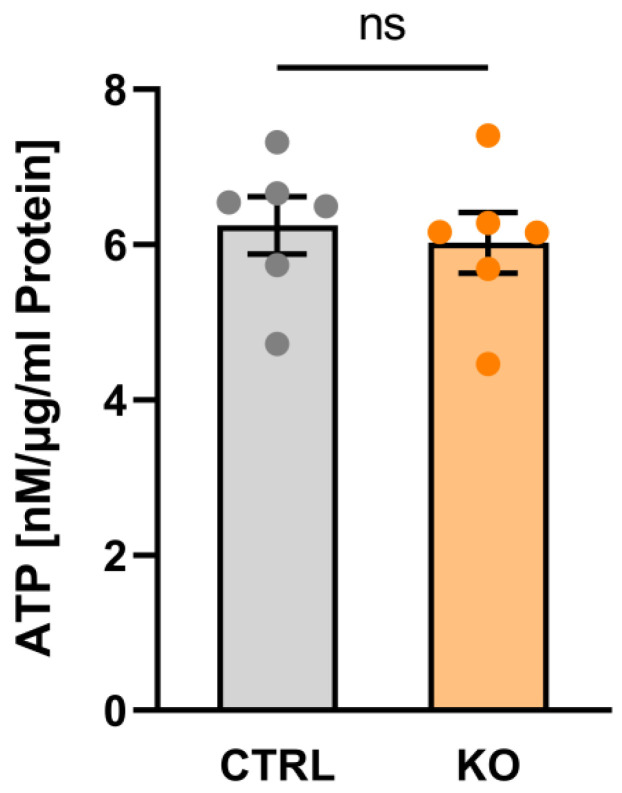
ATP content in human C20 microglia cells. Impact of TSPO deficiency on ATP content. Data are presented as single values as well as mean ± SEM (n = 3) (CTRL 6.246 ± 0.368 vs. KO 6.025 ± 0.389; *p* = 0.4848, Mann–Whitney U test). ns indicates a non-significant difference.

Within a living cell, ATP is generated via two main pathways, i.e., glycolysis and the oxidative phosphorylation system (OxPhos). The overall ATP content in the C20 TSPO−/− and control cells was assessed using a luciferase-based bioluminescence assay. Interestingly, we did not detect a significant difference in overall ATP content in TSPO−/− vs. control C20 cells (Figure 6).

**Figure 7:**
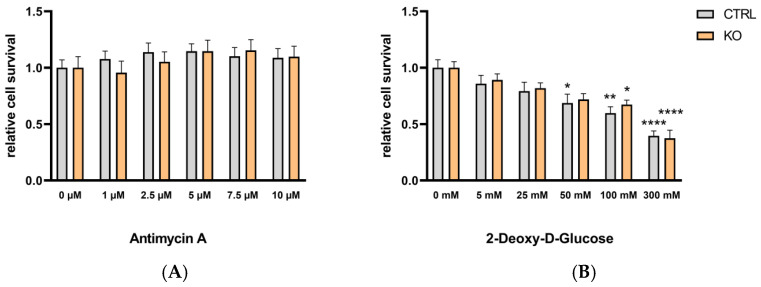
The effect of antimycin A and 2-deoxy-D-glucose on the viability of TSPO-deficient and control C20 microglia cells. (**A**) The effect of antimycin A (1–10 µM) or (**B**) 2-deoxy-D-glucose (5–300 mM) on cell viability was tested using the resazurin assay with TSPO-deficient and control C20 cells. Values represent survival normalized to conditions without antimycin A or 2-deoxy-D-glucose. N = 6, two-way ANOVA, and Tukey’s multiple comparisons test (for data and *p* values, see supplementary Tables S1 and S2; stars indicate significance).

To investigate the effects of TSPO deficiency on the two ATP-generating pathways, glycolysis and OxPhos, we isolated the metabolic pathways by treating the cells for 24 h either with antimycin A (0–10 µM) as an inhibitor of complex III to block oxidative phosphorylation, or with 2-deoxy-D-glucose (0–300 mM) as an inhibitor of glycolysis (by inhibiting the glucose-6-phosphate isomerase). The viability of TSPO-deficient and control cells was assessed by performing resazurin cell viability assays. We found that antimycin A (up to 10 µM) had no effect on the viability of TSPO−/− or control C20 cells, whereas 2-deoxy-D-glucose reduced the viability of C20 cells in a dose-dependent manner, demonstrating their stronger dependency on glycolysis for energy production. However, the viability of the C20 cells was not different in TSPO-deficient or control cells (Figure 7).

It is known that polarization of peripheral immune cells and microglia to an M1 phenotype is often accompanied by a shift in the cells from oxidative phosphorylation to aerobic glycolysis for energy production [4,7].

**Figure 8:**
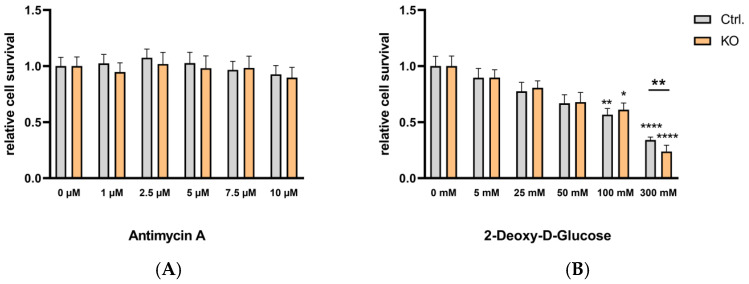
Effect of antimycin A and 2-deoxy-D-glucose on the viability of cytokine-stimulated TSPO-deficient and control C20 microglia cells. (**A**) The effect of antimycin A (1–10 µM) or (**B**) 2-deoxy-D-glucose (5–300 mM) on cell viability was tested using the resazurin assay with TSPO-deficient and control C20 cells. Cells were treated with the cytokine cocktail TNFα, IL-1β, and INFγ (each of 1 ng/mL) for 40 h. Values represent survival normalized to conditions without antimycin A or 2-deoxy-D-glucose. Treating the cells with 300 mM 2-deoxy-D-glucose revealed a significantly different viability of TSPO−/− and control cells (*p* = 0.0026, t = 3991, df = 10, and unpaired *t*-test). For means and *p* values, see supplementary Tables S1 and S2. Stars indicate significance.

To investigate if and how the metabolic profile of TSPO-deficient and control C20 microglia cells is affected by cytokine treatment-induced polarization, we used a cocktail of TNFα, IL-1β, and INFγ (each of 1 ng/mL) [27] and challenged the cells for 16 h (see supplementary Figure S1 and supplementary Tables S1 and S2) and 40 h (Figure 8 and supplementary Tables S1 and S2). Similar to the baseline condition, antimycin A had no effect on C20 viability in cytokine-activated microglia. The dose-dependent reduction of viability by 2-deoxy-D-glucose treatment was evident after 16 h (see supplementary Figure S1 and supplementary Tables S1 and S2) and 40 h (Figure 8 and supplementary Tables S1 and S2) of cytokine treatment. Interestingly, 300 mM 2-deoxy-D-glucose revealed significantly different viability of TSPO−/− and control cells after a 40 h cytokine challenge.

To characterize the impact of cytokine treatment on C20 microglia and to verify the transition to the activated state, we analyzed the mRNA expression levels of TNFα, IL1β, IL6, IL8, IL12A, and NOS2 at basal and cytokine-treated (16 h) conditions.

**Figure 9:**
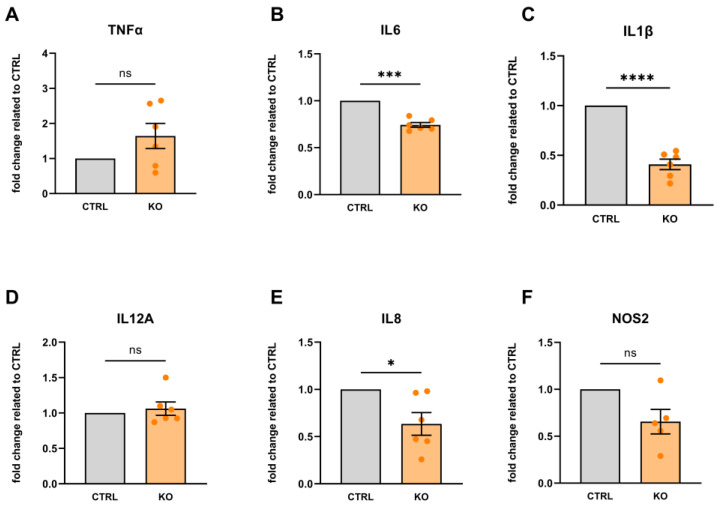
Cytokine mRNA expression in TSPO-deficient human C20 microglia cells normalized to TSPO-expressing control cells (under basal/unstimulated conditions). (**A**) TNFα: 1.642 ± 0.358, *p* = 0.1330; (**B**) IL6: 0.744 ± 0.025, *p* = 0.0002; (**C**) IL1β: 0.409 ± 0.053, *p* < 0.0001; (**D**) IL12A: 1.062 ± 0.094, *p* = 0.5404; (**E**) IL8: 0.634 ± 0.119, *p* = 0.283; and (**F**) NOS2: 0.655 ± 0.130, *p* = 0.0571. N = 6, Welch’s corrected *t*-test. Stars indicate significance. ns indicates a non-significant difference.

Under unstimulated/basal conditions, we found that TSPO-deficient C20 cells showed lower expression of IL1β, IL6, and IL8 mRNA, whereas the expression of TNFα, IL8, and NOS2 was not significantly different from C20 controls (Figure 9). These data demonstrate that TSPO deficiency alters the expression profile of pro-inflammatory cytokines in human C20 microglia cells.

**Figure 10:**
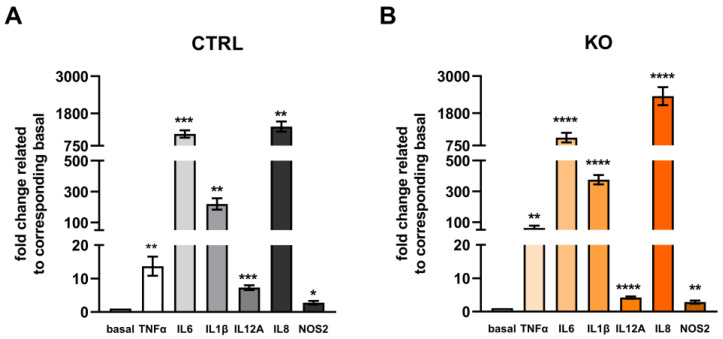
Cytokine mRNA expression in TSPO-deficient human C20 microglia cells and TSPO-expressing control cells after treatment with a cytokine cocktail (16 h). Data represent fold changes in expression related to their unstimulated/basal condition: control (**A**) or TSPO−/− cells (**B**). N = 6, Welch’s corrected *t*-test. For data and *p* values, see supplementary Table S3. Stars indicate significance.

Challenging the C20 cells with a cytokine mixture stimulated the mRNA expression of all cytokine species investigated in both control and TSPO-deficient C20 microglia cells (Figure 10). Looking at the expression profiles within the groups, IL8, IL6, IL1β, and TNFα were the most responsive cytokine genes.

**Figure 11:**
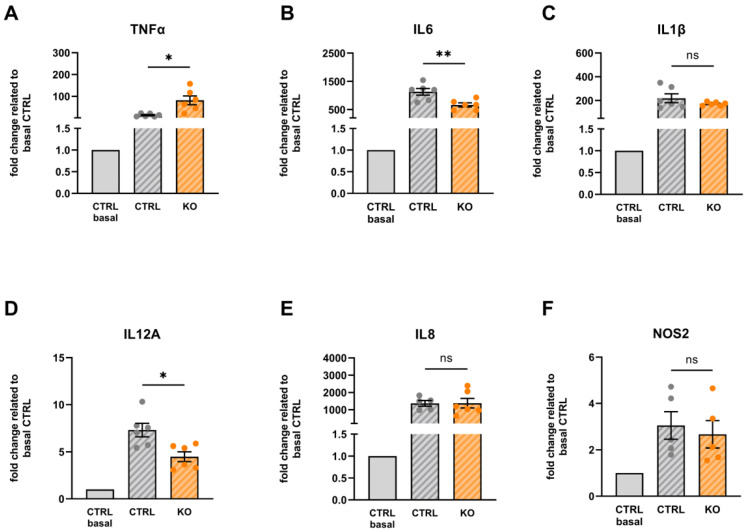
Cytokine mRNA expression in TSPO-deficient and TSPO-expressing human C20 microglia cells after treatment with the cytokine cocktail. The data are related to the unstimulated control of the TSPO-expressing C20 control (gray). (**A**) TNFα: 13.71 ± 2.845 vs. 81.66 ± 20.32, *p* = 0.02; (**B**) IL6: 1129 ± 119.4 vs. 662.7 ± 72.15, *p* = 0.0098; (**C**) IL1β: 219.5 ± 36.70 vs. 172.5 ± 6.305, *p* = 0.2599; (**D**) IL12A: 7.301 ± 0.720 vs. 4.482 ± 0.521, *p* = 0.0112; (**E**) IL8: 1368 ± 164.3 vs. 1378 ± 280.3, *p* = 0.9782; and (**F**) NOS2: 3.047 ± 0.595 vs. 2.668 ± 0.589, *p* = 0.663. N = 6, Welch’s corrected *t*-test. Stars indicate significance, ns indicates a non-significant difference.

A comparison of the effect of cytokine treatment on cytokine mRNA expression in TSPO−/− and control C20 cells revealed a differential expression of TNFα, IL6, and IL12A (Figure 11). TSPO-deficient microglia showed an increased expression of TNFα mRNA but a lower expression of IL6 and IL12A mRNA.

Expression levels of IL1β, IL8, and NOS2 were not significantly different between stimulated TSPO−/− and stimulated control C20 cells (Figure 11).

## 4. Discussion

The functional role of the highly conserved mitochondrial protein TSPO differs between species as well as between cell types. The use of specific knockout models allows for further investigation of the physiological function of this long known but still enigmatic protein.

In the present study, we aimed to shed further light on the question of the cellular functions of TSPO by investigating the impact of TSPO deficiency on human microglia.

TSPO and VDAC are supposed to take part in a still insufficiently defined structural and functional protein complex [26]. As the major pore in the outer mitochondrial membrane, VDAC is involved in the regulation of the diffusion of respiratory chain substrates such as ADP and ATP, NAD^+^, and NADH, cations such as Ca^2+^ and Mg^2+^, and metabolites such as glucose, pyruvate, glutamate, succinate, and citrate [26]. Intriguingly, we observed that TSPO deficiency also affects VDAC1 expression in human C20 cells. Therefore, altered VDAC1 expression may be involved in regulating vital physiological parameters of mitochondrial function. Ongoing studies with VDAC1-deficient or overexpressing cells will clarify how the functional changes observed in TSPO-deficient cells relate to reduced expression of VDAC1. In this context, protein stability assays using cycloheximide will help to analyze the stability of VDAC1 in TSPO-deficient cells [31]. Mutual dependence of TSPO and VDAC expression has also been demonstrated in glioblastoma cancer cells, in which a decline in VDAC expression led to a reduced expression of TSPO [32].

In the present study, we detected significantly higher Ca^2+^ levels both in the cytosol and mitochondria of TSPO-deficient C20 cells compared to the controls. An increased cytosolic Ca^2+^ level in TSPO−/− cells has already been reported by our group [17]. Elevated Ca^2+^ levels may be a consequence of altered Ca^2+^ flux/diffusion or active transport processes, probably affected by altered expression and/or function of the VDAC in the outer mitochondrial membrane, the mitochondrial calcium uniporter (MCU) in the inner mitochondrial membrane, Ca^2+^-exchangers and transporters (ATPases) in the ER (SERCA), or the plasma membrane [24]. Ca^2+^ is a second major messenger within a cell and regulates a variety of processes and cascades affecting ion homeostasis and the OxPhos [33,34].

Our data did not show an effect of TSPO expression on the mitochondrial membrane potential, indicating that the integrated activity of the electron transport chain, the translocation of protons, and the parallel reflux of protons via the ATP synthase, which determine the MMP, are not affected. However, it has already been shown that the impact of TSPO-expression on the MMP is variable and dependent on the species, cell type, and preparation [16,17,35].

The mitochondrial genome exists as multiple copies per cell, with a varying range depending on cellular function, and increased copy numbers in cells that have a higher demand for ATP, like muscle cells or neurons [36]. In the present study, we found a significant reduction in the mtDNA copy number in TSPO-deficient cells. An increased elimination of mitochondria due to increased autophagy as a consequence of ubiquitination and degradation of mitochondrial proteins facilitated by the reduced expression of VDAC in TSPO−/− cells may contribute to these findings [24]. Moreover, a reduced mtDNA has already been shown to negatively affect respiratory capacity, induced by lowered expression levels of proteins involved in the OxPhos [37]. This reduction of oxidative capacity is in line with our earlier studies on TSPO-deficient human C20 cells, in which we observed reduced basal and maximal oxygen consumption rates when compared to the controls [17] and [38].

The total amount of ATP seemed to be lower, although not significantly reduced, in our TSPO-deficient human C20 cells. However, using primary microglia cells isolated from TSPO knockout mice, Yao and coworkers detected a significantly lower ATP level in TSPO-deficient cells, indicating that the reduction of OxPhos in primary mouse TSPO-KO cells led to reduced ATP levels without sufficient compensation through increased glycolytic energy production [6].

Basically, microglia, like most other cell types, express the full complement of gene products required for both glycolytic and oxidative metabolism. Evidence suggests that microglia increase aerobic glycolysis and decrease respiration when activated by various stimuli. Mitochondrial function, glucose availability, and glycolytic rate influence pro-inflammatory gene expression at both transcriptional and post-translational levels [39,40]. A switch in the metabolic and energy-producing pathways from oxidative phosphorylation to glycolysis in microglia or other macrophages may be associated with the polarization of the cells towards the M1 pro-inflammatory state [7,38].

The metabolic profile of our C20 microglia cells already shows a more glycolytic phenotype. It is unclear whether this could be due to a kind of adaptation to a cancer-like state [41,42]. We found a stronger dependence on glycolytic activity than on OxPhos, since 2-deoxy-D-glucose dose-dependently reduced the viability (cell survival) of both TSPO-deficient and control cells, whereas antimycin A did not affect the viability of either group up to a concentration of 10 µM. The highest concentration of 2-deoxy-D-glucose (300 mM) differentially lowered the viability in TSPO-deficient and control cells, indicating that, under these conditions, TSPO had a stronger effect on the glycolytic pathway [22,43]. It has already been reported that TSPO deficiency inhibits microglial activation and impairs mitochondrial function. The authors found that TSPO deficiency significantly inhibited microglial activation induced by LPS or IL-4. Moreover, TSPO deficiency decreased the mitochondrial membrane potential and ATP production. Analysis of cellular energy metabolism showed that TSPO deficiency suppressed mitochondrial oxidative phosphorylation (OXPHOS) and glycolysis, resulting in microglial overall metabolic deficits [6,21].

To investigate the dependence of pro-inflammatory microglial activation on TSPO expression and function, we analyzed the mRNA expression levels of the inflammatory mediators TNFα, IL1β, IL6, IL8, IL12A, and NOS2 at basal/unstimulated conditions and after challenging the cells with a cytokine mixture consisting of TNFα, IL1β, and INFγ [27]. Under basal as well as activated conditions, we found decreased expression levels of IL1β, IL6, IL8, IL12A, and NOS2 mRNA in the TSPO-deficient cell lines. Interestingly, TNFα mRNA expression levels were increased in the TSPO−/− cells at basal conditions and showed even higher levels in cytokine-treated TSPO-deficient cells.

The reduced mRNA expression of pro-inflammatory mediators in C20 TSPO−/− is in line with recent studies reporting similar observations in primary mouse microglia and mouse BV-2 cells, where TSPO depletion led to decreased mRNA expression levels of IL1β, IL6, and iNOS [6]. The authors also described that TSPO-deficient primary microglial cells do not polarize toward an M2 phenotype, suggesting that the involvement of TSPO in microglial activation is not limited to promoting only pro-inflammatory phenotypes. However, findings deviating from this were also reported. Pozzo and co-worker used a C20 TSPO-knockdown model with reduced TSPO expression levels and challenged the cells with LPS as an inflammatory stimulus [44]. The authors reported increased IL8 and decreased IL4 expression in the TSPO knockdown cells in response to LPS stimulation. In contrast to the TSPO knockdown cells, we found a reduced expression of IL8 in TSPO-KO cells in response to stimulation with a cytokine cocktail.

The observed increased TNFα mRNA expression could be explained as a compensatory mechanism in C20 TSPO−/− cells, as TNFα is known to be a downstream modulator of IL1, IL6, and IL8 expression via the NFκB pathway [45], a pathway that is suggested to be itself negatively regulated by TSPO [46]. In addition, the response to stimuli and the function and action of TSPO vary between cell types and species, as evident, for example, in the different responses to stimulation with LPS. Activation of cells did not induce increased TSPO expression in human microglia but did so in rodent microglia [47].

The attenuated response to pro-inflammatory activation may also be related to the alteration in Ca^2+^ homeostasis of the TSPO−/− C20 cell line. A study in mouse microglia reported that changes in activation status were associated with an increase in intracellular Ca^2+^ concentration [48]. The authors also suggested a reduced capacity for Ca^2+^-mediated signal transduction as a side effect of the increase in free cytosolic Ca^2+^, which could affect gene transcription, ion channels, and elements of the cytoskeleton. In this context, it would be of interest to study the Ca^2+^ balance of TSPO-depleted C20 microglia and their controls after their activation.

## 5. Conclusions

Overall, our results show that, in addition to TSPO-dependent modulation of mitochondrial activity and bioenergetic functions, TSPO is also involved in polarizing human C20 microglia toward a pro-inflammatory phenotype upon activation. The TSPO-deficient cells show an attenuated response to activating stimuli in terms of mRNA expression of IL1β, IL6, IL8, IL12A, and NOS2. The increased mRNA expression of TNFα under basal and activated conditions in knockout cells might constitute a compensatory mechanism for counteracting decreased levels of other inflammatory mediators.

## Figures and Tables

**Table 1:** Primer sets used for PCR, sequencing, and QRT-PCR.

Primer Name	Forward Primer (5′-3′)	Reverse Primer (5′-3′)
TSPO-ex1	GAGGTGGCTTTGAGGAGTGA	GCAGGGTTGTCCAAGTTTTC
TSPO-ex2	CTGGAAATGCGTTCACTCAG	GCCTGGAGAAGACCCTCTGT
TSPO-ex3	GAAGCACTGCCAATGTGCTA	GCTTCGTGTGGGTTTTCCTA
TSPO-ex4	AGTTGGGCAGTGGGACAG	GCAGATCCTGCAGAGACGA
TNFA	CCCCAGGGACCTCTCTCTAA	CAGCTTGAGGGTTTGCTACA
IL1B	CTGTCCTGCGTGTTGAAAGA	TGAAGACAAATCGCTTTTCCA
TSPO IL6 IL12A NOS2 CXCL8	TCTTTGGTGCCCGACAAAT CTCAGCCCTGAGAAAGGAGA CATGCTGGCAGTTATTGATGA GTATCCTGGAGCGAGTGGTG TGCGCCAACACAGAAATTAT	GGTACCAGGCCACGGTAGT AGGTTGTTTTCTGCCAGTGC TCAAGGGAGGATTTTTGTGG GACCCAGTAGCTGCCACTCT TGAATTCTCAGCCCTCTTCAA
HPRT1 Mt-TL1 B2M	TTGCTTTCCTTGGTCAGGCA CACCCAAGAACAGGGTTTGT TGCTGTCTCCATGTTTGATGTATCT	ATCCAACACTTCGTGGGGTC TGGCCATGGGTATGTTGTTA TCTCTGCTCCCCACCTCTAAGT

## Data Availability

Data is contained within the article and supplementary material.

## Supplementary Materials

The following supporting information can be downloaded at: https://www.mdpi.com/article/10.3390/cells12060954/s1, Figure S1: Effect of Antimycin A and 2-deoxy-D-glucose on viability of cytokine-stimulated TSPO-deficient and control C20 microglia cells; Table S1: Effect of Antimycin A on viability of cytokine-stimulated TSPO-deficient (KO) and control C20 microglia cells; Table S2: Effect of 2-Deoxy-D-glucose on viability of cytokine-stimulated TSPO-deficient (KO) and control C20 microglia cells; Table S3: Cytokine mRNA expression in TSPO-deficient human C20 microglia cells and control cells after treatment with cytokine cocktail (16 h) and related to unstimulated conditions of control or TSPO-KO cells.
