# Supplementary material for: Impact of Translocator Protein 18 kDa (TSPO) Deficiency on Mitochondrial Function and the Inflammatory State of Human C20 Microglia Cells
Source: Cells. 2023 Mar 21;12(6):954. doi: 10.3390/cells12060954 (PMC10046935; doi:10.3390/cells12060954)
Supplement: Supplementary file 1 [file cells-12-00954-s001.zip › cells-2238748-supplementary.pdf]

Article

Impact of Translocator Protein 18 kDa (TSPO) Deficiency on Mitochondrial Function and the Inflammatory State of Human C20 Microglia Cells

Stefanie Bader †, Thea Würfel †, Tatjana Jahner, Caroline Nothdurfter, Rainer Rupprecht, Vladimir M. Milenkovic and Christian H. Wetzel *

Department of Psychiatry and Psychotherapy, University of Regensburg, 93053 Regensburg, Germany; stefanie.bader@ukr.de (S.B.); thea.wuerfel@web.de (T.W.); tatjana.jahner@ukr.de (T.J.); caroline.nothdurfter@medbo.de (C.N.); rainer.rupprecht@medbo.de (R.R.); vladimir.milenkovic@ukr.de (V.M.M.)

* Correspondence: christian.wetzel@ukr.de; Tel.: +49-941-944-8955

† These authors contributed equally to this work.

SUPPLEMENTARY INFORMATION

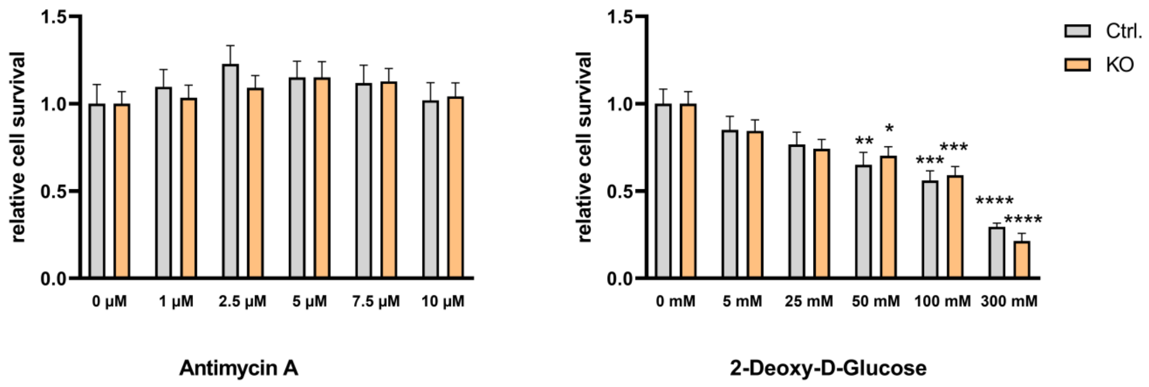

Supplementary Figure S1: Effect of Antimycin A and 2-deoxy-D-glucose on viability of cytokine-stimulated TSPO-deficient and control C20 microglia cells. (A) Effect of Antimycin A (1 – 10 µM) or (B) 2-deoxy-D-glucose (5 – 300 mM) on cell viability was tested using the Resazurin assay with TSPO-deficient and control C20 cells. Cells were treated with cytokine cocktail TNFα, IL-1β and INFγ (each 1 ng/ml) for 16 h. Values represent survival normalized to conditions without Antimycin A or 2-deoxy-D-glucose. Means and p values see supplementary table S1 and S2.

Supplemtary Table S1: Effect of Antimycin A on viability of cytokine-stimulated TSPO-deficient (KO) and control C20 microglia cells (Viability assessed with Resazurin assay)

Cell line and condition	Antimycin A concentration	Mean and SEM	P-value compared to baseline at 0 µM
Ctrl. basal	0 µM	1.000 ±0.069	-
Ctrl. basal	1 µM	1.078 ±0.070	>0.9999
Ctrl. basal	2.5 µM	1.139 ±0.081	0.9917
Ctrl. basal	5 µM	1.146 ±0.066	0.9870
Ctrl. basal	7.5 µM	1.102 ±0.078	0.9995
Ctrl. basal	10 µM	1.008 ±0.081	0.9999
KO basal	0 µM	1.000 ±0.098	-
KO basal	1 µM	0.956 ±0.103	>0.9999
KO basal	2.5 µM	1.053 ±0.088	>0.9999
KO basal	5 µM	1.147 ±0.098	0.9869
KO basal	7.5 µM	1.154 ±0.095	0.9805
KO basal	10 µM	1.098 ±0.093	0.9996
Ctrl. 16h activated	0 µM	1.000 ±0.110	-
Ctrl. 16h activated	1 µM	1.097 ±0.099	>0.9999
Ctrl. 16h activated	2.5 µM	1.228 ±0.105	0.8178
Ctrl. 16h activated	5 µM	1.150 ±0.094	0.9893
Ctrl. 16h activated	7.5 µM	1.118 ±0.102	0.9986
Ctrl. 16h activated	10 µM	1.019 ±0.102	>0.9999
KO 16h activated	0 µM	1.000 ±0.070	-
KO 16h activated	1 µM	1.034 ±0.072	>0.9999
KO 16h activated	2.5 µM	1.091 ±0.070	0.9999
KO 16h activated	5 µM	1.150 ±0.090	0.9890

KO 16h activated	7.5 µM	1.128 ±0.073	0.9971
KO 16h activated	10 µM	1.042 ±0.077	>0.9999
Ctrl. 40h activated	0 µM	1.000 ±0.077	-
Ctrl. 40h activated	1 µM	1.024 ±0.081	>0.9999
Ctrl. 40h activated	2.5 µM	1.075 ±0.077	>0.9999
Ctrl. 40h activated	5 µM	1.026 ±0.098	>0.9999
Ctrl. 40h activated	7.5 µM	0.966 ±0.076	>0.9999
Ctrl. 40h activated	10 µM	0.926 ±0.079	>0.9999
KO 40h activated	0 µM	1.000 ±0.082	-
KO 40h activated	1 µM	0.947 ±0.082	>0.9999
KO 40h activated	2.5 µM	1.018 ±0.103	>0.9999
KO 40h activated	5 µM	0.982 ±0.109	>0.9999
KO 40h activated	7.5 µM	0.984 ±0.104	>0.9999
KO 40h activated	10 µM	0.897 ±0.091	0.9995

Supplementary Table S2: Effect of 2-Deoxy-D-glucose on viability of cytokine-stimulated TSPO-deficient (KO) and control C20 microglia cells (Viability assessed with Resazurin assay)

Cell line and condition	2-Deoxy-D-Glucose concentration	Mean and SEM	P-value compared to baseline at 0 mM
Ctrl. basal	0 mM	1.000 ±0.0708	-
Ctrl. basal	5 mM	0.859 ±0.0731	0.8190
Ctrl. basal	25 mM	0.793 ±0.0786	0.4263
KO basal	0 mM	1.000 ±0.054	-
KO basal	5 mM	0.891 ±0.054	0.9819
KO basal	25 mM	0.819 ±0.047	0.6296
KO basal	50 mM	0.719 ±0.051	0.0752
Ctrl. 16h activated	0 mM	1.000 ±0.083	-
Ctrl. 16h activated	5 mM	0.850 ±0.077	0.8464
Ctrl. 16h activated	25 mM	0.767 ±0.070	0.2522
KO 16h activated	0 mM	1.000 ±0.069	-
KO 16h activated	5 mM	0.844 ±0.063	0.8088
KO 16h activated	25 mM	0.742 ±0.053	0.1385
Ctrl. 40h activated	0 mM	1.000 ±0.087	-
Ctrl. 40h activated	5 mM	0.895 ±0.083	0.9960
Ctrl. 40h activated	25 mM	0.775 ±0.080	0.5332
Ctrl. 40h activated	50 mM	0.668 ±0.076	0.0668
KO 40h activated	0 mM	1.000 ±0.089	-
KO 40h activated	5 mM	0.897 ±0.070	0.9966
KO 40h activated	25 mM	0.806 ±0.062	0.7387
KO 40h activated	50 mM	0.678 ±0.086	0.0668

Supplementary Table S3: Cytokine mRNA expression in TSPO-deficient human C20 microglia cells and control cells after treatment with cytokine cocktail (16 h) and related to unstimulated conditions of control or TSPO-KO cells

	Control mean	Control P-value	KO mean	KO P-value
**TNFα**	13.71 ±2.845	0.0066	62.70 ±14.62	0.0014
**IL6**	1129 ±119.4	0.0002	1007 ±155.2	<0.0001
**IL12A**	7.301 ±0.7202	0.0003	4.233 ±0.3540	<0.0001
**IL1β**	219 ±36.70	0.0019	375.3 ±29.78	<0.0001
**IL8**	1368 ±164.3	0.0011	2348 ±291.6	<0.0001
**NOS2**	2.776 ±0.5568	0.0243	2.851 ±0.4841	0.0065
